# Exploring Health Informatics in the Battle against Drug Addiction: Digital Solutions for the Rising Concern

**DOI:** 10.3390/jpm14060556

**Published:** 2024-05-23

**Authors:** Shakila Jahan Shimu, Srushti Moreshwar Patil, Ebenezer Dadzie, Tadele Tesfaye, Poorvanshi Alag, Gniewko Więckiewicz

**Affiliations:** 1 Department of Health Informatics, Harrisburg University of Science and Technology, Harrisburg, PA 17101, USA; sshimu@my.harrisburgu.edu; 2 SMBT Medical College, Dhamangaon, Nashik 422403, India; srushtipatil2000@gmail.com; 3 School of Clinical Medicine, Inner Mongolia University for the Nationalities, Tongliao 028000, China; ebenezer_dadzie@yahoo.com; 4 CareHealth Medical Practice, Addis Ababa 9023, Ethiopia; ttadele500@gmail.com; 5 Psychiatry Department, Texas Tech University Health Sciences Center, Lubbock, TX 79430, USA; poorvanshi.alag@ttuhsc.edu; 6 Department of Psychiatry, Faculty of Medical Sciences in Zabrze, Medical University of Silesia, 40-055 Katowice, Poland

**Keywords:** drug addiction, substance abuse, electronic health records, Prescription Drug Monitoring Program

## Abstract

Drug addiction is a rising concern globally that has deeply attracted the attention of the healthcare sector. The United States is not an exception, and the drug addiction crisis there is even more serious, with 10% of adults having faced substance use disorder, while around 75% of this number has been reported as not having received any treatment. Surprisingly, there are annually over 70,000 deaths reported as being due to drug overdose. Researchers are continually searching for solutions, as the current strategies have been ineffective. Health informatics platforms like electronic health records, telemedicine, and the clinical decision support system have great potential in tracking the healthcare data of patients on an individual basis and provide precise medical support in a private space. Such technologies have been found to be useful in identifying the risk factors of drug addiction among people and mitigating them. Moreover, the platforms can be used to check prescriptions of addictive drugs such as opioids and caution healthcare providers. Programs such as the Prescription Drug Monitoring Program (PDMP) and the Drug and Alcohol Services Information Systems (DASIS) are already in action in the US, but the situation demands more in-depth studies in order to mitigate substance use disorders. Artificial intelligence (AI), when combined with health informatics, can aid in the analysis of large amounts of patient data and aid in classifying nature of addiction to assist in the provision of personalized care.

## 1. Introduction

Addiction is a chronic, relapsing disease that causes functional alterations in the brain’s networks related to stress and self-control. Long after a person stops using the addictive substance, those changes might persist [1]. Addiction has pervasive impact on various aspects of life, including physical health, mental well-being, relationships, legal status, work, and finances, thus affecting the overall quality of life. Americans lose more than 700 billion dollars annually due to enhanced medical treatment costs, criminality, and diminished productivity due to the use and abuse of prescription drugs, nicotine, and illicit drugs [2,3,4]. In 2018, almost 70,000 deaths were attributed to overdoses of prescription and illicit drugs [4,5].

Since drugs have the most harmful and potentially fatal effects on both the user and others around them, they are typically the first and most prominent thing that comes to mind when someone thinks of addiction. Around 19.7 million adults in the US went through a period of substance abuse in 2017 [6]. Furthermore, over 23 million adult Americans have battled with substance abuse issues. Remarkably, it is said that 10% of adults in their lifetime have experienced a drug-use disorder, but 75% of this population have been found to have never received any treatment [6]. 

Individuals with addiction frequently have one or more comorbid medical conditions, such as lung or heart disease, stroke, cancer, or mental health disorders. Accidental drug overdose has been found to be a major cause of mortality among persons below the age of 45 years. In the US, drug overdose deaths account for over 70,000 deaths per year and the National Centre for Drug Abuse Statistics (2020) has reported that the yearly rate of increase in overdose deaths is 4.0% [7].

The co-occurrence of mental illness and drug use is common. Addiction may lead to mental health issues like psychosis, depression, or anxiety. In other situations, drug use—especially in those with particular vulnerabilities—may cause or exacerbate mental health conditions [8]. Additionally, one in ten HIV cases are related to drug injection. According to Cone et al. (2015), injection drug use can result in endocarditis and cellulitis and is a significant contributing factor in the spread of hepatitis C [9]. It is crucial to acknowledge the availability of FDA-approved medications for opioid-use disorder, and utilizing these in conjunction with behavioral interventions aids individuals in their recovery processes. However, detoxification alone, without further care, usually results in a return to drug use [10]. Given the current increase in drug addiction cases in the US, it is essential to incorporate other interventions in order to prevent and manage addiction effectively.

The use of digital and information technology in healthcare has grown significantly during the first two decades of the twenty-first century. Through data management, personalization, innovative interventions, clinician support, and improved treatment accessibility, information technology offers opportunities for the enhancement of healthcare [11,12]. Health information technology is improving in the US as a result of healthcare reform and a greater focus on performance monitoring. According to Wisdom et al. (2010), these technologies can reduce cost, save time, and improve quality. Increased incentives, data, and implementation guidelines can help improve the facility of health IT in drug addiction treatment [13]. While health informatics’ role in cost-cutting and preventive care has received much attention, its potential for treating addiction remains unexplored. Electronic health records (EHRs), telemedicine, and clinical decision support systems (CDSS) are examples of platforms that show great promise for tracking drug use and providing individualized clinical interventions [12]. Electronic health records (EHR), a clinical decision support system (CDSS), and hospital information management systems (HIM) can aid in the detection of wrong prescriptions and overdose prescriptions of drugs, leading to the prevention of addiction. Moreover, machine learning (ML) algorithms, through brain imaging, behavioral kinematics, and memory analysis, aid by providing insights as to substance use and its associated disorders. 

This review entails a description of the current crisis scenario of drug addiction in the US and the relevant ongoing treatment strategies. This study focusses on how health informatics can be utilized to prevent, monitor, and treat patients with drug addiction, helping them to achieve positive outcomes. The novelty of the review lies in exploring this new side of health informatics in managing a global crisis.

A thorough review of the literature was undertaken across prominent research databases such as PubMed, Scopus, and Google Scholar. Given the focus of this article on computers and the modern world, only articles published after 2001 were included. We specifically considered full-length research articles, systematic reviews, and narrative reviews for analysis, while excluding case reports, case series, letters to the editor, and commentary articles. Two authors screened the databases separately for articles by using the title/abstract method. The search process was meticulous and comprehensive, utilizing keywords such as drug addiction, substance abuse, health informatics, electronic medical records, and electronic health records. Figures were generated using BioRender.com (accessed on 5 January 2024).

## 2. The Growing Drug Addiction Crisis

Drug addiction presents a burgeoning crisis in the US, posing a threat not only to the physical well-being of the youth, but also to their mental health. Given the longstanding nature of this issue, it is imperative to examine past policies while formulating new strategies to address it.

### 2.1. Current Status of Drug Addiction in the US and Related Regulations

Drug addiction has long been a concern in the United States. According to the National Epidemiologic Survey on Alcohol and Related Conditions, men, white people, Native Americans, and those who were single or divorced were more likely to suffer from a drug-use disorder. A higher risk also applied to younger people and those with lower incomes and educational levels [13]. Table 1 represents the 2020 statistics for the US population aged 12 or older as to addiction to various addictive drugs, and their health consequences. 

The Controlled Substances Act (CSA) of 1970 designated many opioids (fentanyl, hydromorphone, morphine, and oxycodone, among others) as Schedule II drugs, and they are currently approved for use in medicine as a form of treatment in the United States, albeit with stringent limitations. According to Chou et al. (2009), the CSA has classified opioids as possessing a highly addictive nature, one that may lead to critical psychological and physical dependency [22].

### 2.2. The Growing Opioid Epidemic 

**Figure 1:**
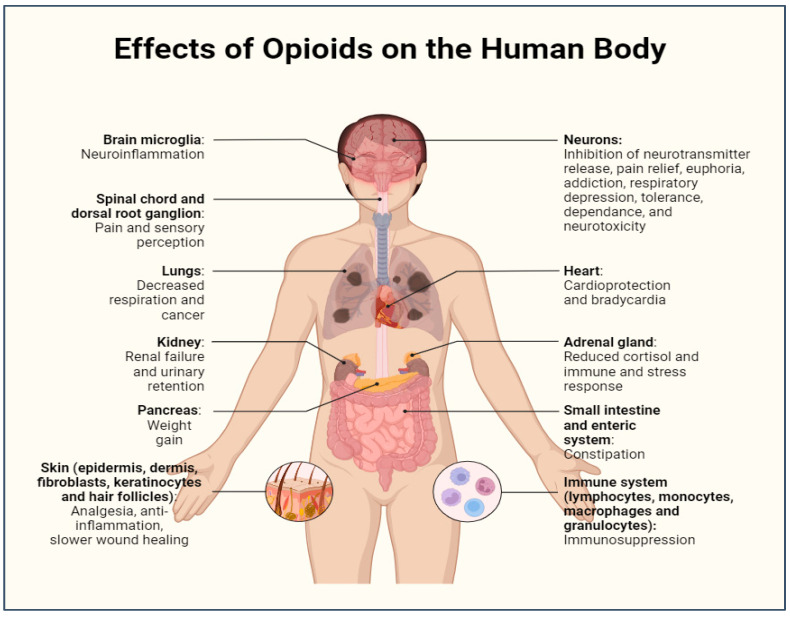
Effects of opioid addiction on the various systems of the human body. https://app.biorender.com/illustrations/65c9b7c73e5b86ada6bdc65d?slideId=39b528df-f03a-4593-a240-a47e2ef7540d (accessed on 5 January 2024).

Opioids reduce pain by slowing signal transmission through the central nervous system. This causes the brain to reduce the sense of pain exponentially [23]. Opioids cause the neurotransmitter dopamine to be released, which is what actually causes physiological and psychological dependence and has given rise to the street term “dope”. Although this is the main goal of the medication, it also causes other physiological effects, including nausea, lack of appetite, and euphoria due to the increased release of dopamine [16]. The overall physical side effects of opioids on the human body are depicted in Figure 1. 

Physical dependence, a medical term often used interchangeably with addiction, can develop within a few days of taking opioids, depending on the dosage and duration of use [24]. Because more and more people are becoming addicted to opioids, the number of prescriptions for these drugs has increased, which has led to an increase in addiction rates. As a result, people are frequently prescribed opioids for a much longer duration than necessary. In 2014, 10.3 million people reported using prescription opioid drugs for purposes other than those for which they were prescribed, or, alternatively stated, 10.3 million people reported taking opioids solely for the euphoric high they produced [25]. The most concerning trends were the 153% increase in emergency room visits related to prescription opioid misuse or abuse between 2004 and 2011, as well as the more-than-quadrupling of patient admissions to substance use treatment programs between 2002 and 2012 [26]. Most alarmingly, in progressing from 1.5 to 5.9 deaths per 100,000 people, morbidity and death from opioid overdoses increased by nearly a factor of four between 2000 and 2014. The DHHS designated opioid addiction as a major public health epidemic in 2014 after over 165,000 prescription-opioid-related deaths were reported [26].

### 2.3. The Mental Health Consequences and Social Impact

Addiction to substances is linked to numerous immediate and long-term health consequences. They can differ based on the medication type, dosage, and frequency of use, as well as the individual’s overall health [27]. In general, drug use and dependence can have a wide range of consequences, affecting nearly every organ in the human body. Moreover, they can lead to difficulties with memory, focus, and decision-making which impede day-to-day functioning. Changes in mood, thought, or behavior are often indicative of mental disorders [28]. They can make daily tasks challenging and hinder a person’s capacity to work or perform well in school, engage with family, and carry out other important life-tasks [29]. 

There are various ways that drugs can impact mental health. Drug use can cause long-term mental health issues. Cannabis use on a regular basis may raise your risk of depression or anxiety [30]. Increased levels of cannabis use have also been connected to the development of schizophrenia or psychosis [31]. People may experience anxiety and depression following the use of stimulants. Stimulants like cocaine have the potential to exacerbate pre-existing psychological issues and schizophrenia [32]. “Magic mushrooms” and other hallucinogenic substances can induce frightening or upsetting flashbacks and a sense of detachment from your surroundings. Combining medication with alcohol or other drugs can pose serious risks, including those of potential harm or fatality. The present-day mind is more knowledgeable about the potential consequences of combining various medications.

### 2.4. Associated Risk Factors of Drug Addiction

**Figure 2:**
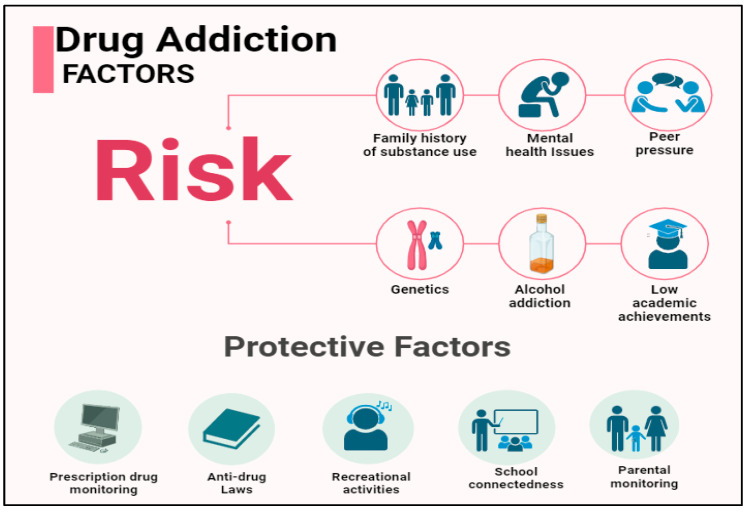
Protective and risk factors associated with drug addiction among individuals. https://app.biorender.com/illustrations/65c9bf623838038a35e0a3a6?slideId=212350a7-bd36-4a9a-baa9-8e46347669a1 (accessed on 5 January 2024).

The chance of becoming addicted to drugs varies from person to person, just as with other illnesses and disorders, and there is no one factor that can predict whether someone will develop an addiction. Generally speaking, the likelihood that using drugs will result in drug use and addiction increases with an individual’s number of risk factors. Conversely, protective factors lower an individual’s risk. Environmental and biological factors can act as risk and protective factors (Figure 2). The majority of people who use drugs at some point in their lives do so without ever developing a substance-use disorder, despite the fact that numerous studies have identified factors that predict the likelihood of disorders associated with substance use. For instance, it is estimated that 46.9% of Americans have used marijuana at some point in their lives, but only 9.9% of US adults will experience a drug-use disorder at some point in their lives [33,34]. Any age, gender, or socioeconomic background can develop addiction. The likelihood and rate at which addiction develops can be influenced by various factors.

#### 2.4.1. Record of Addiction within a Family

Drug addiction has been found to be more prevalent in certain families and is probably associated with a higher risk in some cases due to genetics. You run a higher risk of becoming addicted to drugs if you have a blood relative, like a parent or sibling, who is addicted to alcohol or drugs [35]. Meier et al. (2016) created a cumulative risk index, for instance, by adding up the presence of nine risk factors that are associated with childhood and adolescence: being male, having a lower socioeconomic status in the family, having a family history of drug addiction, having depression as a child, being exposed to substances at an early age, and frequently using alcohol, tobacco, and cannabis as a teenager [35].

#### 2.4.2. Mental Health Disorder

Addiction to drugs is more common in those with psychological issues like depression, attention-deficit/hyperactivity disorder, or post-traumatic stress disorder. One of the most well-researched indicators of the likelihood of developing a substance use disorder as an adult is receipt of a mental health diagnosis early in life. Attention-deficit/hyperactivity disorder, conduct disorder or oppositional defiant disorder, and depression diagnosed in childhood or adolescence were linked to an increased risk for adult addiction, according to a meta-analytic review [36].

#### 2.4.3. Peer Pressure, Lack of Family Involvement and Early Use

Peer pressure plays a significant role in the decision to start using and abusing drugs, especially for young people [37]. Addiction risk factors include challenging family circumstances, a lack of attachment to parents or siblings, and inadequate parental supervision. Drug use during adolescence can alter the brain’s development and raise the risk of developing a drug addiction [38].

## 3. Health Information Management and Addiction

With the increasing incidence of drug addiction among individuals in the US, it is time for health information technology to be explored, allowing it to attain its full benefits in monitoring and preventing addiction.

### 3.1. Health Informatics and Various Platforms

Health information technology (IT) includes a wide array of devices, programs, and networks that facilitate patient-centric care or self-management among patients [39]. Clinical alerts, computerized order entry, clinical decision support systems, electronic prescribing and test results, patient decision support, administrative and financial systems, and other electronic exchanges of health information are a few of the instances of applications related to health informatics [40]. Information on the clinical and behavioral conditions of clients, as well as information about finances, regulations, and other mandated reporting, are all included in health information. 

Hospital settings that have to handle data from several departments (like radiology, pharmacy, and intensive care) and various types of quantitative data (like lab results and prescription orders) will undoubtedly benefit from these essential capabilities [41]. Health informatics improves the quality of preventive care by providing providers with patient-centric alerts on issues like vaccine schedules. Quality indicators can be measured by aggregating data from multiple patients. After entering data from hand-filled forms, staff members can immediately increase productivity [12]. By permitting electronic invoicing, billing schedules can be optimized, and communication delays caused by paper invoices being mailed or faxed can be minimized. According to Ekstrom and Johansson (2019), health IT can also lower medical errors brought on by a lack of communication, incomplete information, or illegible handwriting [42].

These advantages are seen in health IT systems that track a large number of patients. These systems allow large hospitals to take advantage of economies of scale that might not be available to treatment programs, which typically have fewer patients [41]. Enhanced vaccination rates and decreased post-operative infections are two examples of preventive care measures for which decision support systems have been linked to adherence to protocol-based care [40]. Harpaz et al. (2013), for instance, developed interventions to lessen the frequency of adverse drug events by using electronic medical records to identify them [43]. However, Liu and colleagues (2013) discovered that because of data fragmentation and a poor human–machine interface, putting in place a computerized order-entry system increased the risk of medication errors [44].

### 3.2. Applications of Health Informatics in Drug Addiction Management

There are studies available on the cost-effectiveness of substance use treatment and the cost of implementing health IT. Research examining these problems in medical facilities and physician practices has usually focused on how health IT has affected service utilization [45]. On the other hand, medical interventions are less common in substance use treatment programs (29 percent test for HIV and 21 percent test for other STDs); instead, the majority of programs (99% offer individual therapy and 96% group therapy) focus primarily on psychosocial interventions [14]. Health IT offers organizational opportunities in addition to information administration. It can assist in monitoring client health statistics over time, giving a clear picture to support the management of addiction.

**Figure 3:**
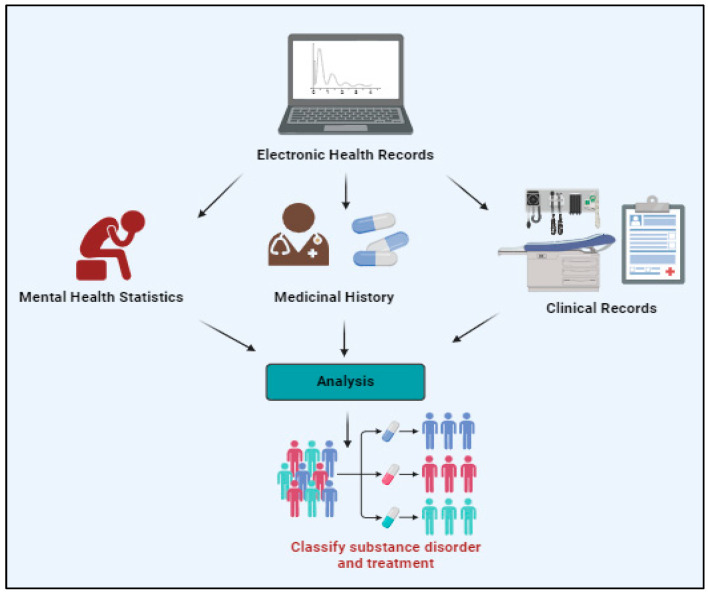
Role of electronic health records in the management and treatment of drug addiction. https://app.biorender.com/illustrations/65c9d39fe1ecacdd7c66b249 (accessed on 5 January 2024).

Electronic health record (EHR) systems facilitate the retrieval of comprehensive patient data, encompassing medical history, prescriptions, and records of substance use treatment. Data on substance use disorders (SUDs) can be integrated into electronic health records (EHRs) to support coordinated care and guarantee continuity between healthcare settings [46] (Figure 3). Remote addiction treatment and support services can be conveniently provided through telemedicine platforms and mobile health apps. Individuals with addiction can receive medication-assisted treatment, peer support networks, and counselling via secure communication channels and virtual consultations [47]. 

Predictive analytics is another tool that health informatics uses to identify people who may return to use or develop drug-use disorders. In order to lower high-risk prescribing behaviors, opioid medication use, and mortality rates, Valdes et al., 2023 set out to use the recently developed Opioid Risk Stratification Tool to identify people who might be at risk for abusing opioids. They also sought to investigate the effects of implementing a mailing and engagement intervention to this population and their prescribers [48]. According to Valdes et al. (2023), there was a higher decline in the number of people in the intervention group who also had prescriptions for benzodiazepines and opioids [48]. In addition, Prescription Drug Monitoring Programs (PDMPs) also keep track of prescriptions for controlled substances in order to spot possible misuse or diversion. By enabling real-time data analysis, alerts for questionable prescribing patterns, and interoperability with EHRs for smooth information exchange, health informatics tools improve the functionality of prescription drug monitoring programs (PDMPs) [49].

### 3.3. Prescription Drug Monitoring Programs (PDMPs) and Their Effectiveness

Prescription drug monitoring programs (PDMPs) are presently in use in a majority of the American states to identify providers who overprescribe opioids and to discourage patients from doctor shopping, which is the practice of routinely obtaining duplicate prescriptions for opioids. According to Yokell et al. (2012), PDMPs include a plethora of data on demographic trends, prescription drug-use patterns, overdoses, and poisonings. These data can be analyzed to identify shifts in prescribing practices and patterns that are influencing the changing trends [50].

Incorporating the first significant health information management tool to be used for both diagnosing and preventing prescription drug addiction, this strategy was historic. Despite the fact that the PDMP was first created for law enforcement [51], prescribing physicians were able to view a patient’s medicinal history in real time, and, on occasion, at the time of an urgent necessity. According to a 2012 study by Dormuth et al., PharmaNet, the PDMP databases used in Canada, led to a sharp decline in the prescription of opioids [52]. 

The frequency of PDMP use by doctors in pain management, psychiatry/behavioral health, internal medicine, and dentistry was studied by Hildebrand et al. in 2014 [53]. The study’s findings showed that while not all prescribers use a PDMP consistently, patients needing pain management frequently received the same prescriptions. In contrast, doctors with different specializations mainly used PDMP for first-time clients, or for patients who appeared to be seeking drugs. 

A PDMP utilization survey was carried out by Irvine et al. (2014) on 1065 Oregon physicians who were currently employed in pain clinics, emergency medicine, and primary care [51]. Around 95% of the prescribers who took part in the survey at the time said they had used the PDMP while treating a patient who may have been using illicit drugs. As for first-time patients, who are not suspected of having drug-use disorders, about half of the prescribers surveyed said they utilized the PDMP. Referrals for psychological help or addiction therapy were made 54% of the time. 

**Figure 4:**
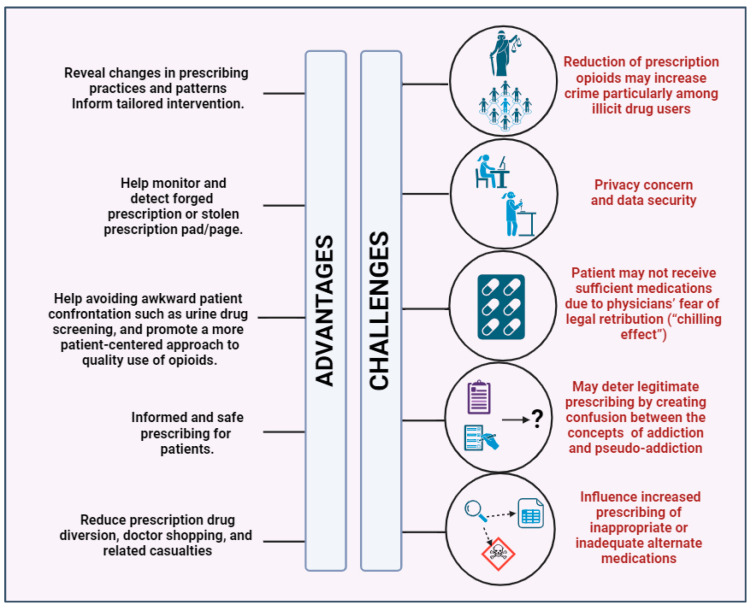
Advantages and challenges related to the use of a prescription drug monitoring system in controlling prescribing addictive medications. https://app.biorender.com/illustrations/65c9d4930b30aa000ee90ce5?slideId=7e85bb6c-29d1-44fe-a542-a5fb3b97cb51 (accessed on 5 January 2024).

In 2014, Islam and McRae revealed that physicians not willing to suggest opioids to specific patients due to concerns about abuse were likely to receive low ratings, which may have an impact on their ability to receive payment and keep their jobs [54]. These studies show that Health IT platforms such as PDMP can aid in identifying patients struggling with drug addiction, even though the PDMP has its own benefits and drawbacks (Figure 4). Further investigation is required to enhance and optimize the methods by which medical practitioners use health informatics to identify and address both suspected and confirmed drug abuse.

## 4. Development of Novel Interventions

Apart from PDMP, there are a few novel interventions that have been developed involving health information technology. These include Drug and Alcohol Services Information Systems and AI-based interventions.

### 4.1. Drug and Alcohol Services Information Systems (DASIS)

In recent years, addiction treatment has been affected globally by the use of health information management. These database structures are often known as Substance Dependency Treatment Information Systems (SDTIS) or Drug and Alcohol Services Information Systems (DASIS), and are health information management systems that gather, process, and disseminate data about addiction [26]. Since substance abuse and addiction have such widespread effects on modern society, the WHO (World Health Organization) ranks drug addiction among the most pressing challenges confronting our generation, along with hunger, criminal activity, and pollution of the environment [55]. This validates the need for these specific databases. 

It is crucial to implement the right care schedules for each patient, just as it is for the prevention and management of any psychological or physical condition. The System Data Set, Minimum Data Set, and Supplementary Data Set are the three subsets of data that the US uses to treat addiction using the DASIS system. The goal of this information system is to give data regarding drug abuse and addiction therapy, including patient demographics, hospitalization and discharge records, and program efficacy metrics [56].

### 4.2. Developing a Collaboration between EHRs and AI

Currently, there is very little computational support available for carrying out extensive research that can measure each of these medications’ effects on an individual basis, whether they are taken alone or in combination. This is mostly because different people respond to these medications differently, which frequently leads to conflicting findings in scientific cohort studies.

Using a socioeconomic status survey dataset from Bangladesh, Shahriar et al., 2019 used Neural Networks, Random Forests, Support Vector Machines, and feature importance to differentiate between people who were addicted and those who were not [57]. Conversely, Dong et al., 2021 employed deep networks to forecast drug addiction, employing the Cerner EMR dataset [58]. While both offer insightful information and showcase the application of state-of-the-art computational technology, neither investigates the potential for creating a patient-level metric that is proportionate to the person’s propensity towards substance use. Ovalle et al., 2021 evaluated the risks of abuse based on four bio-markers: HIV, amphetamines, methamphetamines, and tetrahydrocannabinol (THC, a constituent of cannabis) [59]. They obtained data from social media sites. Additionally, Barenholtz et al. (2020) identified several aspects that can be improved to incorporate computational strategies, including machine learning, into the understanding and management of substance abuse [60]. 

The standardization of data and procedures is one of these elements. Quantifying the impact of addictive prescription drugs on individuals through the use of objective data is one potential solution to these problems [61]. Therefore, it may now be possible to use biological data to quantify substance effects using Electronic Medical Record (EMR) data [62]. Additionally, the use of informatics like the SEI can help to advance research and diagnosis related to substance abuse. When the SEI is completed, it could be used in conjunction with the psychometric scores to (i) give the treating physician a more comprehensive and insightful profile of the patient’s substance abuse; and (ii) promote the use of standardized methods and measures, which will advance research on substance use [62].

According to research described in [63], machine learning (ML) algorithms can aid in drug-addiction determination through various factors. Brain-related factors, behavioral phenotypes, and functional differentiation of the brain can express a great deal about disorders. These findings also identify the insights into various research levels, classification techniques, performance measures, challenges, and future directions related to use of ML. Random Forests models are largely used, due to their better performance. 

Through the integration of electronic health records (EHR) in healthcare systems, the details of the journey of a patient through treatment can be easily monitored. The details as to the various prescribed medicines and their doses can be tracked at any point, and through the interoperability of systems, by any other hospital. This can aid in detecting unnecessary opioid prescription and improper dosing.

Moreover, through Clinical Decision support systems (CDSS), dosing can be tracked. Dosing errors account for over 60% of all prescribing mistakes [64]. But through CDSS, the software component can generate a personalized list of recommended dosages for a specific medicine. Moreover, the CDSS can address the problem of duplicate therapy by comparing a newly introduced medication with the active ingredients of drugs in a patient’s profile. If a similarity is detected, the system generates an alert, eliminating the chance of an overdose. This can be beneficial, too, in restricting opioid overdoses. People working at hospitals who are engaged in healthcare management need to have the proper training as to the handling and supervision of these forms of software.

## 5. Challenges in Implying Health Informatics for Drug Addiction

The application of health informatics to drug addiction treatment presents a number of obstacles, despite the possible advantages. It is crucial to protect sensitive patient data, which requires strong data encryption, controlled access, and adherence to privacy laws [39]. Coordinated care and information sharing are hampered by the fragmentation of healthcare data across different systems. To enable data sharing between agencies and providers, standardization and interoperability initiatives are required [65].

Furthermore, unequal access to technology could worsen already-existing healthcare inequalities by restricting the use of health informatics interventions in marginalized communities [39]. Furthermore, there are moral conundrums associated with the use of patient data for surveillance, research, and predictive modelling; thus, procedures for informed consent are required [65].

These health informatics systems need to be carefully designed to keep matters only between the healthcare provider and patient. The software applications need to adhere to high security standards, specifically those involving HIPAA compliance, and need to be blockchain secured for increasing privacy and mitigate data theft. In the revised manuscript we have added this data, as highlighted in [66].

## 6. Conclusions and Future Prospects

The field of health informatics has enormous potential to change how drug addiction is treated in the United States. Healthcare stakeholders can improve prevention efforts, optimize therapy pathways, and encourage sustained recoveries among people who suffer from substance use disorders. To fully utilize health informatics in the fight against the growing problem of opioid and other drug addictions, however, issues of confidentiality, connectivity, and fairness must be resolved [67].

Future initiatives should concentrate on collaboration among healthcare sectors, government bodies, technology developers, and community members to create inventive informatics in order to maximize the impact of health informatics on drug addiction management [39]. Furthermore, it is crucial to carry out thorough investigative research to determine the efficacy of health information technology in the treatment of drug addiction [67]. Drug addiction treatment can undergo a revolution if data analytics and machine learning algorithms are used to design therapies based on an individual patient’s unique risk profile, treatment preferences, and socioeconomic background.

## Figures and Tables

**Table 1:** Statistics of people in the US (aged 12 or above) affected by substance abuse in 2020, and the associated physical and mental health consequences.

Substance	Estimated Number of People Affected	Physical/Mental Health Consequences	References
Marijuana	5.1% (or 14.2 million)	Alterations in senses, Mood swings, Diminished bodily movement, Difficulty in problem-solving, Decreased memory, Hallucinations, Psychosis	[14,15]
Opioids	1.1% (or 2.7 million)	Falling unconscious, Slow and shallow breathing, Choking, Vomiting, Slower heart rate	[14,16]
Cocaine	0.5% (or 1.3 million)	Weight loss, Damage to cardiovascular system, Risk of stroke, Intracerebral hemorrhage, Ulcerations in the GI tract, Cognitive impairments	[14,17]
Heroin	0.3% (or 902,000)	Hot flashes, Dry mouth, Lack of concentration, Slower heart rate, Coma and Permanent brain damage	[14,18]
Stimulant use disorder	0.2% (roughly 500,000)	Decreased appetite, Anxiety, Jitteriness, Headaches, Weight loss, Insomnia, Psychosis.	[14,19]
Benzodiazepines	2% (5 million)	Respiratory depression, Respiratory arrest, Drowsiness, Confusion, Syncope, Nausea/vomiting, Diarrhea.	[14,20]
Barbiturates	0.2% (or 500,000)	Lack of consciousness, Bradycardia, Difficulty in coordination, Vertigo, Weak muscles	[14,21]

## Data Availability

No new data were created.
